# Effects of temporal properties of facial expressions on the perceived intensity of emotion

**DOI:** 10.1098/rsos.220585

**Published:** 2023-01-11

**Authors:** Yuki Harada, Junji Ohyama, Makoto Wada

**Affiliations:** ^1^ Developmental Disorders Section, Department of Rehabilitation for Brain Functions, Research Institute of National Rehabilitation Center for Persons with Disabilities, Tokorozawa, Japan; ^2^ Faculty of Humanities, Kyoto University of Advanced Science, Kyoto, Japan; ^3^ Human Augmentation Research Center, National Institute of Advanced Industrial Science and Technology, Kashiwa, Japan

**Keywords:** perceived intensity of emotion, autistic trait, emotion, social cognition, pupil size

## Abstract

A series of multiple facial expressions can be temporally perceived as an averaged facial expression in a process known as ensemble perception. This study examined the effect of temporal parameters on the perceived intensity of facial expression in each emotion, and how the effect varies with autistic traits in typically developing people. In the experiment, we presented facial expressions that switched from emotional to neutral expressions, and vice versa, for 3 s. Participants rated the overall perceived intensity of the facial emotions as a whole rather than rating individual items within the set. For the two tasks, a ratio of duration of emotional faces to duration of neutral faces (emotional ratio) and the timing for transitions were manipulated individually. The results showed that the intensity of facial emotion was perceived more strongly when the presentation ratio increased and when the emotional expression was presented last. The effects were different among the emotions (e.g. relatively weak in the anger expression). Moreover, the perceived intensity of angry expressions decreased with autistic traits. These results suggest that the properties and individual differences in the facial ensemble of each emotion affect emotional perceptions.

## 1. Introduction

Facial emotion recognition plays a key role in social communication. In daily situations, individuals interpret others' facial expressions to evaluate their mental state because expressions contain non-verbal social information, such as an individual's emotional state [1] and intention [2]. Facial information strongly influences impression formation [3]. The underlying mechanisms of facial emotion recognition have been investigated in previous studies about sociability [4,5].

Data from automatic analysis of interview scenes suggest that facial expressions change dynamically in daily life [6]. Therefore, understanding the temporal characteristics of facial emotion recognition is important to clarify the role of facial emotion recognition in communication. Emotion recognition in changing facial expressions may relate to ensemble perception, which is the process wherein individuals aggregate multiple visual items, for example, by determining an averaged facial expression based on several individual faces (i.e. a facial ensemble) [7,8]. This facial ensemble has been observed for multiple items that are arranged not only in different spatial locations but also in different temporal positions [9,10]. The facial ensemble (especially the temporal one) is useful for successful communication because facial expressions change dynamically during communicative interactions [6]. Facial changes involve various expressions, for instance, micro-expressions [11], which reveal concealed emotional information [12]. Thus, abstracting facial changes may contribute to mentalizing the emotional state of others.

Furthermore, the ability to recognize changing facial expressions is atypical in individuals with autism spectrum disorder (ASD) [13–16]. ASD is characterized by impaired sociability and sensory, perceptual and cognitive characteristics that differ from those of typically developing persons [17,18]. Kessels *et al*. [13] presented participants with a set of facial expressions that gradually changed from neutral to emotional and asked them to choose the emotion that appropriately described the facial set. Generally, individuals with ASD show poorer performance when identifying fear and disgust expressions, compared with typically developing persons. Reports indicate that people with ASD show atypical responses of attention and pupil sizes toward facial expressions. For example, while typically developing persons fixated on the eyes longer than the mouth, people with ASD did not show these attention patterns [19,20]. Moreover, during the presentation of facial expressions, pupil sizes were larger in infants at high risk of developing ASD than in those with a low risk [21]. Given that pupil sizes are influenced by arousal [22], the atypical emotional recognition in people with ASD would be related to emotional arousal. This idea has been supported by evidence from cognitive neuroscience. For example, the amygdala in persons with ASD has been reported to be unresponsive [23,24] or unable to adapt [25] to facial expressions when compared with typically developing persons. Given that the amygdala is related to facial emotion recognition, especially for negative emotional expressions [26,27], this specific activity would contribute to impairment in the recognition of such expressions in individuals with ASD [28–30]. Since these differences negatively influence communication between persons with and without ASD, investigating how autistic traits influence facial emotion recognition is important for understanding sociability in persons with ASD.

There has been mixed evidence regarding the effect of autistic traits on spatial facial ensembles. For example, Rhodes *et al*. [31] reported diminished facial ensemble perception in individuals with ASD; however, Karaminis *et al*. [32] did not observe such an effect. More recently, Chakrabarty & Wada [33] reported that peripheral face ensembles unconsciously affect the judgement of a facial emotion of a central target, both in participants with typical development and autism spectrum condition. They also observed large individual differences in spatial facial ensembles, especially when individuals intentionally recognized the averaged emotion of five faces. In their experiment, a neutral expression was presented at the centre of a display, while several emotional expressions were presented around the target. Half of the participants with ASD showed a low spatial ensemble perception when asked to abstract emotional expressions. These results suggest that there is significant divergence between facial ensembles and that some cognitive factors may mediate the relationship between autistic traits and facial ensemble perception.

The first purpose of this study was to examine which factors influence the temporal ensemble of a face. In this respect, we focused on how the ratio of presentation time for different facial expressions would potentially influence perception. Daily facial expressions frequently switch between several emotional and neutral expressions in different durations. The presentation time ratio for each facial expression may influence the perceived intensity of the facial emotion. There is indirect evidence underscoring the effects of the emotional ratio, thus extending the duration of facial expression presentations can enhance temporal facial ensembles [9]. However, this effect has only been examined when using expressions of disgust. Since daily communication involves several emotional expressions, such as anger, disgust, fear, happiness, sadness and surprise [1], the interaction between facial emotions and the duration of their presentation may be important. Previous neuroscientific and behavioural research suggests that the time course of emotion processing interacts with facial expressions. For example, negative facial expressions cause the activities of the amygdala [34] and event-related potentials [35] to occur at short latencies. Consequently, anger expressions can be quickly recognized [36,37]. The evidence suggests that negative expressions may be less susceptible to the presentation time ratio. To investigate this, in the present experiment (Task 1), we examined the temporal ensemble of emotions with emotion presentation ratios in the range of a few seconds (3 s), considering that there are differences among emotions. If the presentation time ratio influences the temporal ensemble, the perceived emotional intensity would increase with the duration of emotional faces compared with the duration of neutral faces.

The timing of the presentation of specific emotional expressions could also influence ensemble perception. Emotional items presented at the primacy and recent temporal positions of the stimulus strongly influence impression formation [38]. Fang *et al*. [39] found that an expression presented at the last position strongly influenced impression formation (e.g. competence, warmness and affiliation); however, the authors did not examine the order effects on the temporal ensemble. The purpose of Task 2 is to examine whether primacy/recency effect is strong in the perception of each emotion. If the primacy and/or recency effects occur in the temporal ensemble, the perceived emotional intensity would increase when an emotional expression is presented at the first/last position of the facial sequence.

The second purpose of this study was to examine the effect of autistic traits on temporal ensembles. Previous studies have suggested specific systems of time perception in ASD [40–42]. Particularly, these could mediate atypical temporal ensembles in individuals with ASD. On this point, changes in arousal and attention to facial expressions may be important. As noted above, the research indicates that individuals with ASD have atypical activity in the amygdala [23] and atypical attentional allocation for facial expression [19,20,43]. Thus, the atypical nature may influence the temporal ensemble of individuals with ASD. Therefore, the present study measured pupil sizes and viewing times for facial expressions using an eye tracker to clarify this issue.

We investigated the effects of the ratio of presentation time and onset timing of facial expressions by temporal ensemble tasks. Traditionally, a temporal ensemble task involves viewing multiple facial images presented in a series. These faces were arranged in an order that morphs to show different intensities of emotions. Thus, participants could determine whether a test face was more emotional than the perceived average of multiple faces [9]. However, the morphing-face procedure would not be suitable for investigating the effects of presentation time ratio and onset timing, as the effects of the two temporal factors would be confounded by the intensity of facial emotions. Consequently, this creates challenges in interpreting the results. Therefore, a simplified procedure was used in this study. Specifically, participants viewed serially presented faces that switched between neutral and emotional faces. Then, participants could estimate the perceived intensity of facial emotion averaged across the faces provided.

In this study, we examined the effect of the ratio of presentation time and onset timing of facial expressions on temporal ensemble, and the effect of autistic traits on temporal ensemble, arousal and attentional allocations. In two tasks, after viewing a face that changed between an emotional and a neutral expression, participants were asked to rate the perceived intensity of the facial emotion. For the change, the ratio of emotional expression time to total facial presentation time and the onset timing of emotional expressions were manipulated. Participants' autistic traits were measured by the autism-spectrum quotient (AQ score [44]). Two hypotheses were proposed: (Hypothesis 1) if the emotional ratio and/or onset timing of facial expression influence temporal ensemble then the perceived intensity of the facial emotion would change with the independent variables; (Hypothesis 2) if autistic traits influence temporal ensemble, arousal and attentional allocation, then the AQ score would negatively correlate with the perceived intensity of facial emotion, pupil size and viewing times, respectively.

## 2. Material and methods

### 2.1. Participants

In total, 49 typically developing individuals (23 men and 26 women; mean age = 22.33 years, s.d. = 3.47) participated in the experiment. Of these participants, 36 performed Task 1, which investigated the effect of the emotional expression time ratio to total facial presentation time, whereas 28 performed Task 2, which investigated the effect of the onset timing of emotional expressions. These sample sizes were determined by previous studies that investigated the effect of facial expressions on the observer's emotion [45–47]. All participants performed at least one of the two tasks that assessed the perceived intensity of changing facial expressions. Fifteen participants who agreed to join the experiment performed both tasks. Additionally, all participants performed a pre-test that assessed the facial emotion recognition of static expressions. All participants had normal or corrected-to-normal visual acuity and were naive as to the purpose of the experiment.

### 2.2. Apparatus and stimuli

A desktop personal computer (HP Z230 Tower Workstation) was used to present the stimuli on an LED monitor (HP Pavilion 2311f; 1920 × 1080 pixels; 51° × 29° of visual angle; 60 Hz). This was controlled using Matlab (Mathworks, Natick, Massachusetts, USA) with the Psychophysics Toolbox extensions [48–50]. Since people with ASD show atypical patterns of pupil size [51] and gaze [19,20], participants' eye movements were recorded using EyeLink 1000 PLUS (SR Research, Ottawa, Canada).

We used facial expressions displaying six basic emotions (anger, disgust, fear, happiness, sadness and surprise) and neutral emotion. These were expressed by six actors (three males and three females). In total, 42 pictures of facial expressions [(6: emotional + 1: neutral) × 6: actors] were selected from the facial database of Advanced Industrial Science and Technology [52]. In this database, the emotional valence and arousal for the facial expressions were measured by using the valence (1: *very negative* to 9: *very positive*) and arousal ratings (1: *sleepy* to 9: *aroused*). The valence ratings for the respective expressions were 2.54 for anger, 2.40 for disgust, 3.01 for fear, 7.83 for happiness, 2.95 for sadness and 5.17 for surprise. The arousal ratings for these expressions were 6.71 for anger, 5.88 for disgust, 6.99 for fear, 5.68 for happiness, 4.13 for sadness and 7.95 for surprise.

The Japanese version of the AQ [44] was used to evaluate participants’ autistic traits. This questionnaire (comprising 50 questions) asked participants the extent to which they agreed with social communication situations (1: *disagree* to 4: *agree*).

### 2.3. Tasks

Overall, we performed a pre-test and two main tasks. In the pre-test, emotion recognition of static facial expressions was measured. A trial sequence of pre-test was conducted as follows: after the space key was pressed, a black fixation cross (+) was presented for 1 s. Subsequently, one of the six emotional expressions and its six choices was presented. The participants were asked to select the choice that appropriately described the presented emotional expression. Eye movements were not recorded. After the selection, the next trial began. This task comprised 72 trials: facial emotion (6) × repetitions (12). The order of the facial emotions displayed was randomized within the block.

**Figure 1.**
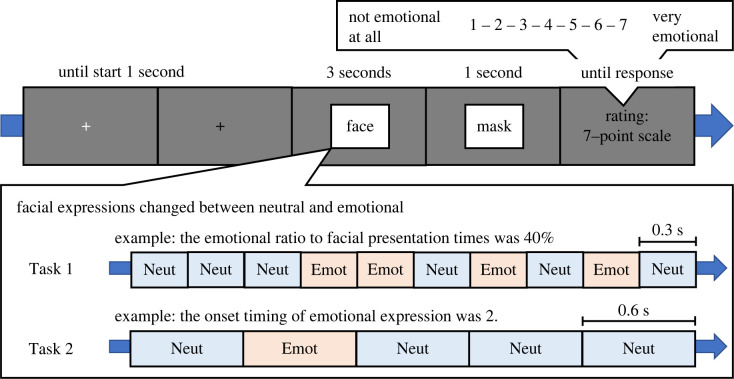
Schematic illustration of a trial sequence in Tasks 1 and 2. In the two tasks, the facial expressions changed between neutral and emotional based on the ratio of emotional expression time to total facial presentation time (Task 1) or onset timing (Task 2). After the facial presentation, participants indicated their perceived intensity of facial emotion on a 7-point scale.

In Task 1, we examined how temporal ensemble was influenced by the ratio of emotional expression time to total presentation time, and how this influence changed with autistic traits. A trial sequence of Task 1 is shown in figure 1. After the start key was pressed, a white fixation cross was replaced with a black fixation cross that lasted for 1 s. Subsequently, a face (7° × 7° of visual angle) was presented for a 3 s period, during which it changed between neutral and emotional expressions. The facial presentation was divided into 10 frames of 0.3 s, and neutral and emotional expressions were presented in randomly selected frames (facial identity was fixed in each trial). The ratio of emotional expression time to total facial presentation time was manipulated in six levels (0, 20, 40, 60, 80 and 100%), which we will refer to as the **‘**emotional ratio’. For example, an emotional ratio of 40% represented emotional expressions in four frames and neutral expressions in the remaining six frames (these onset positions were randomly selected). To assess the effect of emotional expression on arousal and attentional allocations, the participants' eyes were recorded while they were viewing the facial presentation. After the random dot mask (1 s), the participants used a 7-point scale to estimate the overall perceived intensity of facial emotions, as opposed to a part of the presentation (i.e. 1: *not angry* *at all* to 7: *very angry*). After the rating, the next trial began. Task 1 comprised 216 trials: facial emotion (6) × emotional ratio (6) × repetitions (6). The facial emotion was manipulated between blocks, and the order of emotional ratio was randomized within each block.

In Task 2, we examined how the presentation's timing of emotional expression influenced the temporal ensemble and how this influence changed with autistic traits. Task 2 was identical to Task 1 except for different facial presentations (figure 1). The facial presentation was divided into five frames of 0.6 s each. One of the frames depicted the emotional expression, while the others depicted the neutral expression. The timing of the presentation of the emotional expression was manipulated into five positions, which we will refer to as **‘**onset timing’. Task 2 comprised 180 trials: facial emotion (6) × onset timing of emotional expression (5) × repetitions (6). The order of onset timing was randomized within each block.

### 2.4. Procedure

After receiving experimental instructions, the participants provided written informed consent and completed the AQ and empathy/systemizing quotient (EQ/SQ). Subsequently, they sat on a chair and their heads were stabilized with a chin rest. The participants performed the pre-test and either Task 1 or 2 on the same day. Fifteen participants performed the two tasks on separate days.

### 2.5. Analysis

We analysed the effects of emotional ratio and onset timing on the perceived intensity of facial emotion and how these effects changed with AQ scores. The AQ score was calculated according to Wakabayashi *et al*. [44], in which only the **‘***partially agree’* or **‘***agree*’ responses to autistic items and **‘***disagree*’ or **‘***partially disagree*’ responses to inverted autistic items were counted. The participants' AQ scores ranged from 6 to 34. Similarly, EQ and SQ scores were calculated. The D score was calculated according to criteria, in which the standardized EQ score was subtracted from the standardized SQ score. Participants’ D scores ranged from −3.59 to 3.96 (mean = −0.09, s.d. = 1.69). The EQ/SQ/D scores were analysed only to check the distribution of participants' autistic traits.

We also computed pupil size and viewing times of faces. Pupil size was used to evaluate participants’ arousal levels when viewing the faces. There was difficulty analysing pupil sizes obtained from the emotional ratio of 20–80% because these conditions produced variations in pupil sizes due to changes in the physical features of facial images. Therefore, pupil sizes were obtained only from emotional ratios of 0 and 100%. Pupil sizes were calculated from all fixations and irrespective of a specific area of interest. In this analysis, pupil sizes were converted into z scores by using their mean and standard deviation in each participant and experimental block. This is because the baseline of their size was different across participants and blocks. The viewing times of faces were used to evaluate attentional allocation during the facial presentation.

To investigate the effects of emotional ratio (or onset timing) and autistic traits on attentional allocation, we analysed participants' viewing times for the eyes and mouth. In these analyses, three areas of interest (rectangular) were set on the left eye, right eye and mouth for each facial expression. In each trial, the duration of participants’ gaze lingering on these areas of interest was registered as the viewing time.

Before these calculations, the eye movements obtained from eight participants in Task 1 and four participants in Task 2 were excluded based on two criteria: (i) failed calibration of the eye-tracking system, and (ii) extremely inaccurate tracking of the eye. For the first criterion, when the calibrations failed three times, we performed the experiment without recording eye-tracking. This failure occurs frequently when participants are equipped with astigmatic or hard contact lenses. For the second criterion, calculated eye positions obtained from one participant extremely deviated from the actual eye positions (more than 10° of visual angle in half trials). Moreover, we excluded eye-movement data when the eye blinks occurred or data obtained from trials that produced many misdetections of the pupil (more than 25%).

The statistical software R (i.386, 3.6.3) and R studio (v. 1.2.5033) were used to perform the data analyses. For statistical significance tests, linear mixed-effects models (LMMs) were performed using the **‘**lmer’ function in the **‘**lmerTest’ package. In analysing data for Task 1, facial emotion (anger, disgust, fear, happiness, sadness, surprise; categorical variable), emotional ratio (20, 40, 60, 80%; continuous variable) and AQ score (continuous variable) were set as fixed effects. In analysing data for Task 2, facial emotion, onset timing (0–0.6, 0.6–1.2, 1.2–1.8, 1.8–2.4, 2.4–3.0 s; continuous variable) and AQ score were set as fixed effects. In these analyses, the participants and subjects of the face stimuli were set as random intercepts. The final model was determined by the successful convergence and backward stepwise elimination (the ‘step’ function was used). Main effects and interactions were examined by the **‘**anova’ function. Multiple comparisons were performed by using the ‘lsmeans’ function in the ‘lsmeans’ package, in which *p*-values were adjusted with Tukey's method.

## 3. Results

### 3.1. Pre-test: identification of static facial expressions

**Figure 2.**
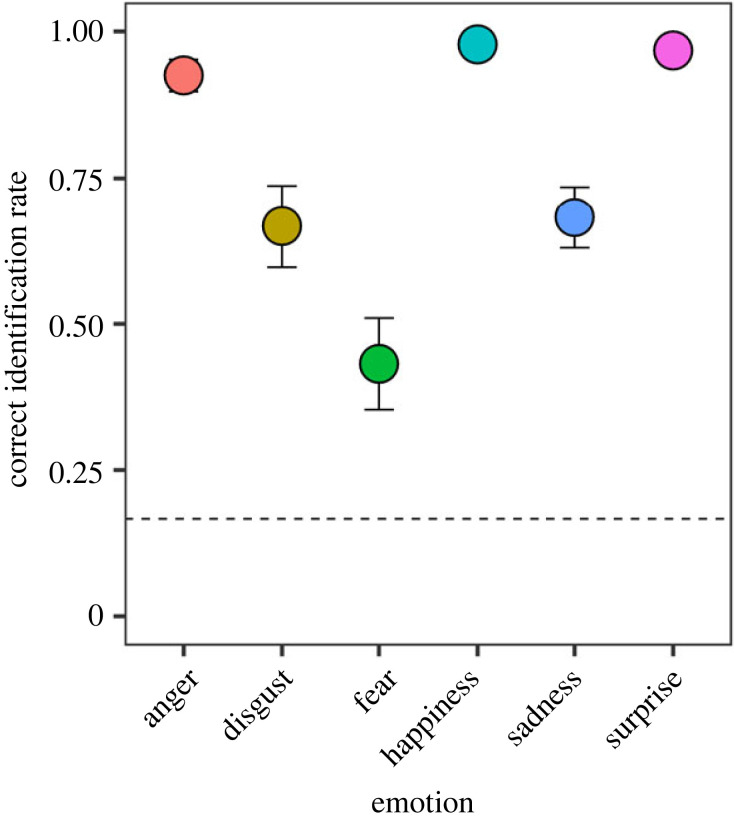
Emotion recognition for static facial expressions. Correct identification was averaged across the participants. The error bars represent 95% confidence intervals. The dotted line represents the chance level.

The identification performances obtained from the pre-test are shown in figure 2. An LMM was performed on the identification performance with the fixed effects of facial emotion (anger, disgust, fear, happiness, sadness and surprise) and AQ. The LMM of the final model revealed that the main effect of facial emotion was significant [*F*_5, 3464_ = 32.355, *p* < 0.0001]. The results of a multiple comparison test are shown in electronic supplementary material, table S1. The LMM also showed a significant interaction between facial emotion and AQ score [*F*_5, 3464_ = 2.585, *p* = 0.024]. A *post hoc* analysis revealed that identification performance for the anger expression was negatively correlated with AQ score (*r* = −0.307, *t* = −2.211, *p* = 0.032), but the identification for the other expressions was not (|*r*|s < .177, |*t*|s < 1.231, *p*s > 0.224). The results show that autistic traits negatively influence the identification of static anger expressions.

### 3.2. Task 1: effect of emotional ratio

#### 3.2.1. Behavioural data

**Figure 3.**
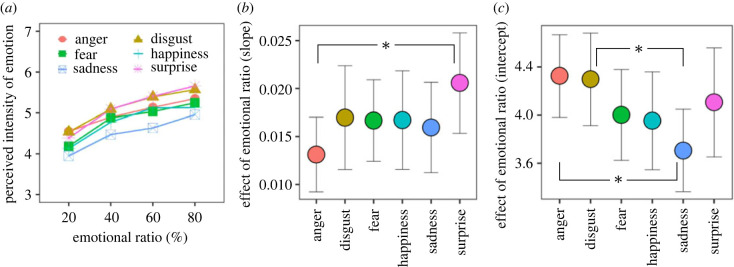
Effect of the ratio of emotional expression time to total facial presentation time on temporal ensemble in Task 1. In this task, participants indicated the perceived intensity of facial emotion for facial expressions that changed between neutral and emotional based on the ratio of emotional expression time to total facial presentation time. (*a*) The perceived intensity of facial emotion averaged across the 36 participants. (*b*) The slope of rating data as a function of emotional ratio. (*c*) The intercept of rating data as a function of emotional ratio. Asterisks represent significant differences between facial emotions, *p* < 0.05.

Figure 3*a* shows the perceived intensity of facial emotion in Task 1, where facial expressions changed between neutral and emotional based on emotional ratios. The data obtained from the 0 and 100% condition of emotional ratio were excluded because these conditions did not change facial expressions (excluded data are shown in electronic supplementary material, figure S1). An LMM of the final model showed significant main effects of facial emotion [*F*_5, 5132_ = 18.139, *p* < 0.0001] and the emotional ratio [*F*_1, 5132_ = 641.262, *p* < 0.0001]. The results of a multiple comparison test are shown in electronic supplementary material, table S2. The results also showed that the two-way interaction for facial emotion × AQ score was significant [*F*_5, 5132_ = 5.208, *p* < 0.0001]. A *post hoc* analysis revealed that the perceived intensity of the anger expression was negatively correlated with the AQ score (*r* = −0.353, *t* = −2.203, *p* = 0.035), but the ratings for the other expressions were not (|*r*|s < 0.305, |*t*|s < 1.866, *p*s > 0.071).

To examine whether the effect of emotional ratio was varied by facial emotion, we calculated the slope and intercept of rating as a function of emotional ratio (figure 3*b,c*). The slope indicates the amount of emotional ratio effect while the intercept shows the effect of facial emotion irrespective of emotional ratio. LMMs were performed on the slope and intercept with fixed effects of facial emotion and AQ score. For the slope, the main effect of facial emotion was significant [*F*_5, 175_ = 2.575, *p* < 0.0282], in which the effect of emotional ratio was significantly smaller for the anger than for the surprise expression (*t* = −3.546, *p* = 0.0066). For the intercept, the main effect of facial emotion was significant [*F*_5, 175_ = 4.589, *p* = 0.000586], in which the intercept was significantly larger for the anger than for the sadness expression (*t* = 4.045, *p* = 0.0011) and significantly larger for the disgust than for the sadness expression (*t* = 3.864, *p* = 0.0021).

In Task 1, the number of emotional expressions varied across trials even with the same emotional ratio. To examine the effect of the number of emotional expressions, an LMM was performed on the rating data with the fixed effects of number of emotional expressions, facial emotion and AQ. The number of emotional expressions was not manipulated. Thus, certain combinations of the number of facial changes and emotional ratio were rarely observed (e.g. the number of facial changes was one with an emotional ratio of 40%). Therefore, the data obtained from the conditions were excluded from this analysis. The LMM of the final model showed that the number of emotional expressions significantly increased in the perceived intensity of facial emotion [*F*_1, 4855_ = 24.813, *p* < 0.0001]. Moreover, the main effect of emotion and the interaction for facial emotion × AQ were significant [*F*_5, 4921_ = 18.478, *p* < 0.0001; *F*_5, 4921_ = 5.385, *p* < 0.0001].

These behavioural data suggest that both the ratio and the number of emotional expressions influence temporal ensemble. The results also show that autistic traits negatively affected the perceived intensity of anger expressions that changed over time. Since this was consistent with the results obtained from the pre-test, individuals with higher autistic traits presumably evaluate angry expressions as low, irrespective of static or changing expressions. Moreover, slope and intercept analyses indicated that the effect of the ratio on the perceived intensity of emotion interacts with facial expressions. In particular, the perceived emotional intensity for anger expressions was strong even when the emotional ratio was small and less susceptible to the emotional ratio.

#### 3.2.2. Eye data

**Figure 4.**
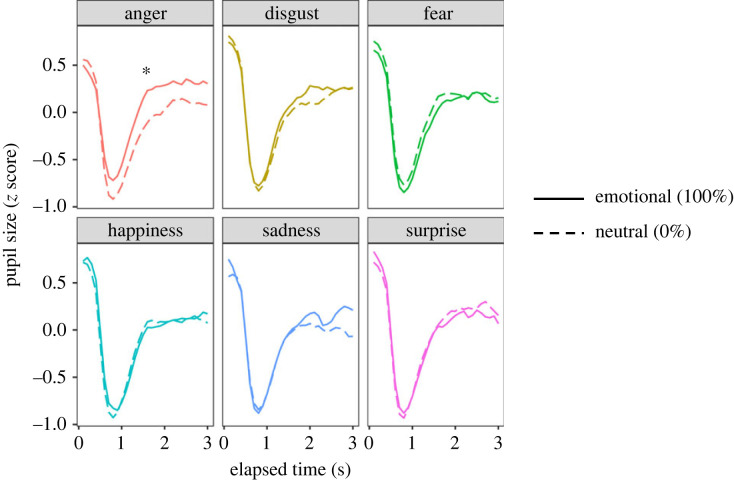
Changes in pupil size during the facial presentation in Task 1. The pupil size as a function of the elapsed time. The curved lines represent the means of pupil sizes across the participants. The asterisk represents the significant difference between the emotional (100% ratio condition) and neutral (0% ratio condition) (*p* < 0.05).

Since pupil size increases with emotional arousal [22], we analysed pupil sizes to examine the effects of facial emotion and autistic traits. In Task 1, we extracted the emotional ratios 0 and 100% conditions and calculated the means of normalized pupil sizes as a function of elapsed time (figure 4). For each facial emotion condition, LMMs were performed on the averaged pupil size with fixed effects of emotional ratio (0 and 100%) and AQ score. Facial emotion was not entered as a fixed effect because the pupil size was converted into a Z score for each facial emotion. The LMM of the final model revealed that the pupil sizes were significantly larger when viewing the anger expression than at viewing the neutral expression [*F*_1, 317_ = 8.040, *p* = 0.00487]. For the other emotional expressions, neither any main effect nor interaction was significant (*F*s < 3.619, *p*s > 0.0581). These results show that arousal levels increased during the presentation of anger expressions.

**Figure 5.**
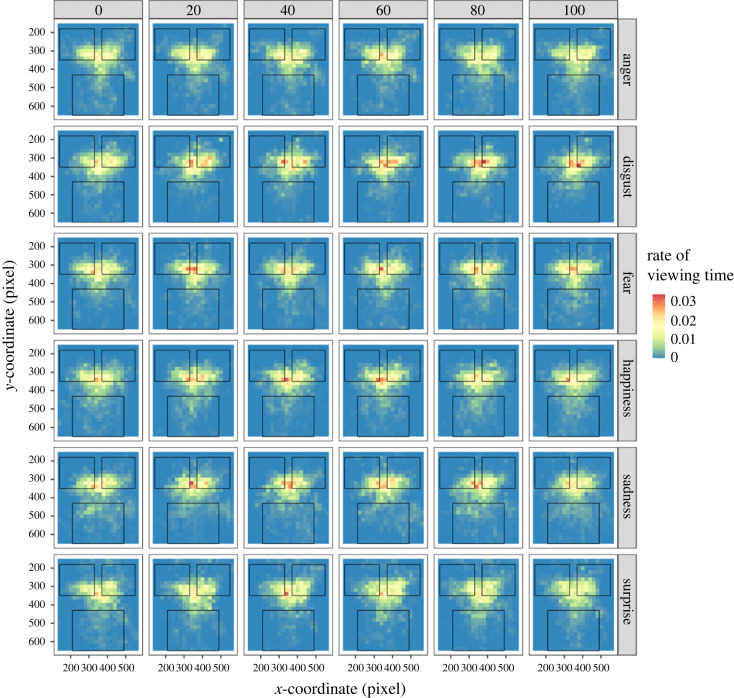
Attention allocation for facial expressions. The colour of the heat map represents the rate of viewing time in each area. The upper left and right squares in each panel show the area of the eyes, and the lower square shows the area of the mouth.

To examine the effect of changing facial emotional expression on attentional allocation, we computed the viewing times for the eyes and mouth of the faces displayed in Task 1 (figure 5 and electronic supplementary material, figures S2A and S2B). For the viewing time of eyes and mouth, the LMMs of the final model produced some significant results. For the viewing time of eyes, the main effect of facial emotion was significant [*F*_5, 3728_ = 6.381, *p* < 0.0001]. The results of a multiple comparison test are shown in electronic supplementary material, table S3. The LMM also showed a significant interaction for facial emotion × AQ score [*F*_5, 3728_ = 6.324, *p* < 0.0001]. A *post hoc* analysis revealed that the viewing time on eyes of disgust expression was negatively correlated with the AQ score (*r* = −0.424, *t* = −2.384, *p* = 0.025). The viewing times for the eyes for the other emotional expressions were not correlated (|*r*|s < 0.229, |*t*|s < 1.202, *p*s > 0.240). For the viewing time of the mouth, the main effects of facial emotion and emotional ratio were significant [*F*_5, 3727_ = 5.559, *p* < 0.0001; *F*_1,3727_ = 5.806, *p* = 0.0160]. The results of a multiple comparison test are shown in electronic supplementary material, table S4. Moreover, the interaction for facial emotion × AQ score was significant [*F*_5, 3727_ = 6.213, *p* < 0.0001]. A *post hoc* analysis revealed that the viewing time on a mouth of any emotional expression was not correlated with the AQ score (|*r*|s < 0.24, |*t*|s < 1.27, *p*s > 0.22).

### 3.3. Task 2: effect of onset timing

#### 3.3.1. Behavioural data

**Figure 6.**
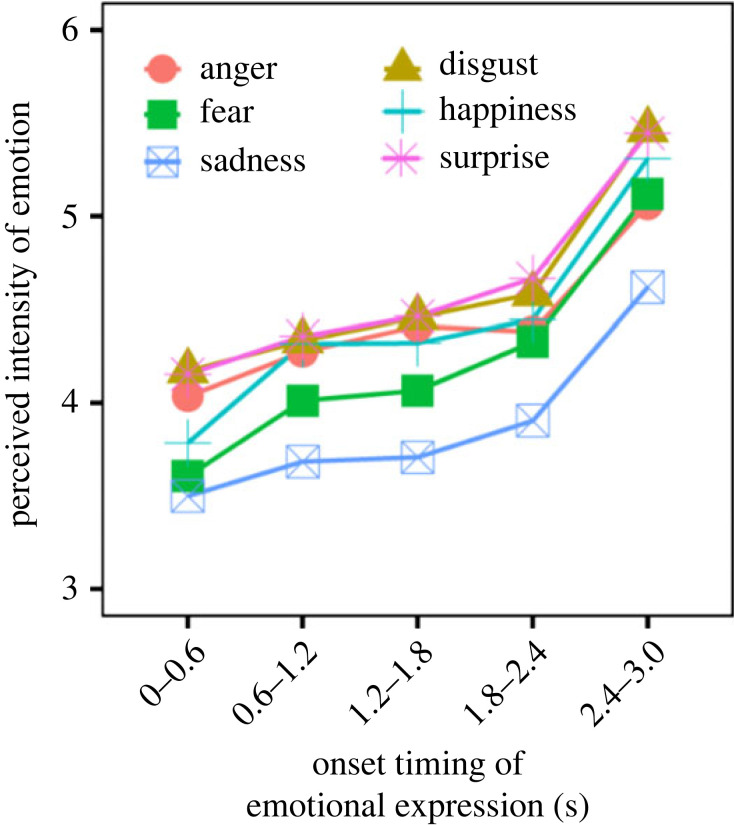
Effect of onset timing of facial expressions on the temporal ensemble in Task 2. In this task, participants indicated the perceived intensity of emotion for faces that changed between neutral and emotional based on onset timing conditions.

Figure 6 shows the perceived intensity of facial emotion in Task 2, where facial expressions changed between neutral and emotional based on the onset timing conditions. An LMM of the final model showed significant main effects of facial emotion [*F*_5, 4990_ = 5.053, *p* = 0.000127] and onset timing [*F*_1, 4990_ = 614.350, *p* < 0.0001]. The results of a multiple comparison test are shown in electronic supplementary material, table S5. The results also showed significant interactions for facial emotion × onset timing [*F*_5, 4990_ = 2.527, *p* = 0.0271] and facial emotion × AQ [*F*_5, 4990_ = 3.670, *p* = 0.00257]. For the former interaction, the effect of onset timing was significantly lower for the anger condition than for the fear and happiness conditions (*b* = 0.118, s.e. = 0.0393, *t* = 3.012, *p* = 0.00261; *b* = 0.102, s.e. = 0.0393, *t* = 2.603, *p* = 0.00926). For the latter interaction, the perceived intensity of emotion was not significantly correlated with AQ score in each facial emotion (|*r*|s < 0.365, |*t*|s < 2.001, *p*s > 0.056). These results suggested that the intensity of emotion is strongly perceived by the recency of the expressions. Moreover, the intensity of anger was relatively unsusceptible to the recency of facial expressions. This may partially stem from anger perception at 0.0–0.6 s position, which is larger than happiness, fear and sadness. The relatively larger perception of anger is consistent with the intercept analysis in Task 1.

#### 3.3.2. Eye data

Pupil sizes obtained from Task 2 were not analysed because facial expressions changed in all facial emotion conditions. In this condition, it was difficult to detect the effect of facial emotion because pupil sizes were significantly influenced by the changes in stimulus luminance.

**Figure 7.**
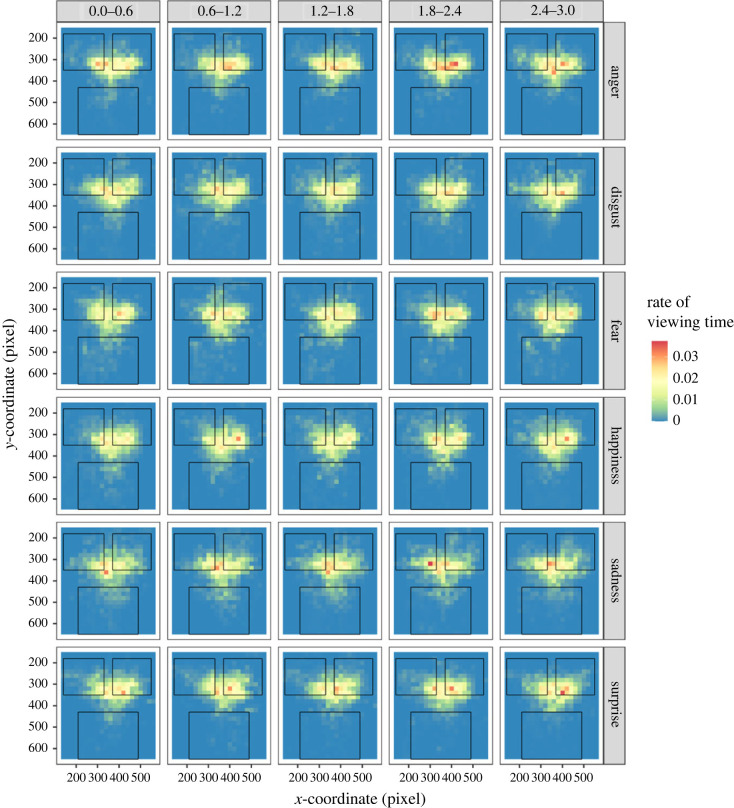
Attention allocation for facial expressions. The colour of the heat map represents the rate of viewing time in each area. The upper left and right squares in each panel show the area of eye movement, and the lower square shows the area of a mouth.

To investigate the effect of the onset timing of expressions on attentional allocation, we computed participants' viewing times on the eyes and mouth of faces displayed in Task 2 (figure 7, electronic supplementary material, figures S3a and S3b). For the viewing times of eyes, an LMM of the final model showed significant main effects of facial emotion [*F*_5, 4084_ = 18.755, *p* < 0.0001] and onset timing [*F*_1, 4084_ = 8.182, *p* = 0.00425]. The results of a multiple comparison test are shown in electronic supplementary material, table S6. For viewing times for the mouth, an LMM of the final model showed significant main effects of facial emotion [*F*_5, 4085_ = 25.819, *p* < 0.0001] and onset timing [*F*_1, 4085_ = 7.118, *p* = 0.00766]. The results of a multiple comparison test are shown in electronic supplementary material, table S7. Moreover, the interaction for facial emotion × AQ was significant [*F*_5, 4085_ = 17.502, *p* < 0.0001], but a *post hoc* analysis revealed a non-significant correlation between the viewing times and AQ in each facial emotion (|*r*|s < 0.317, |*t*|s < 1.603, *p*s > 0.123).

### 3.4. Relationship to the valence and arousal

A new analysis was conducted to discern the role of arousal. We examined the effects of arousal and valance of facial expression on the behavioural data of the pre-test, Task 1 and Task 2. In these analyses, LMMs were conducted for the response of each task using fixed effects of arousal and valence (plus emotional ratio in Task 1 or onset timing in Task 2; see electronic supplementary material, Analysis S1). Consistent with the above explanation, the results showed that the correct identification in the pre-test and rating data in Tasks 1 and 2 were significantly influenced by arousal (and/or valence). Similarly, the effects of emotional ratio and onset timing on the perceived intensity of emotion were significant. Furthermore, the effect of emotional ratio (figure 3*b*) was not influenced by arousal or valence. Moreover, if arousal and valence explain the anger-specific effect, this effect could yield fear expressions because the two ratings of anger expression are similar to those of fear expression. This suggests that the anger-specific effect cannot be explained using arousal or the valence of facial expressions alone.

## 4. Discussion

The present experiment was conducted to investigate (i) how temporal ensemble is influenced by the ratio of emotional expression time and the onset timing of emotional expressions, and (ii) how autistic traits influence temporal ensemble, arousal and attentional allocations. Consistent with Hypothesis 1, this study found that the presentation time and onset timing of facial expressions affected the temporal ensemble. Moreover, the *post hoc* analysis demonstrated the effect of the number of changes in facial expressions on the perceived intensity of emotion. These results suggest that visual statistical systems use relative presentation times of the facial expressions provided and the weight averaging calculations according to the timing of facial changes during temporal ensembles.

The finding for Task 1 suggests two interpretations: (i) emotional ratio is used to perceive temporal ensemble for any facial expressions of basic emotion, but (ii) the effect of emotional ratio varies across basic emotions. The former idea is reasonable because this is consistent with the effect of exposure times of each facial expression on temporal ensemble [9]. However, the latter interpretation would require more specific explanations, such as why the effect of emotional ratio on the temporal ensemble of anger expression is low. This may be related to the processing of negative emotional information. Anger expressions were quickly recognized [36,37] and triggered psychophysiological responses (e.g. skin conductance) even when they were subliminally presented [53]. This knowledge indicates efficient processing of negative emotional information even with brief exposures. This is important from an evolutionary perspective, in which information related to perceived threats would be detected more easily [54]. In this perspective, the lower effect of the anger ratio would be effective to avoid potential threats (e.g. aggressions), and it may influence social communication. In fact, as discussed later, individual differences in the recognition of angry faces were associated with autistic traits.

The results of Task 2 show that the recency effect occurs in the context of temporal ensemble perception. This may be interpreted by the attentional explanation. An item presented more recently would be emphasized because it receives more attention [38]. Contrary to the recency effect, the primacy effect does not occur in the temporal ensemble. This may be related to the facial presentation duration in our tasks. The primacy effect has been reported to relate to the attention paid to each item [55]. In the present experiment, participants’ attention would be sustained for the whole facial presentation because its duration was relatively short (3 s). Considering that mutual face-gazing is performed for a short time (approx. 2.2 s [56]), it can be presumed that the recency effect on emotion perception occurs in daily communication.

Hypothesis 2 was partially supported by behavioural data. We found an interaction between the facial emotions and AQ score, in which the perceived intensity of angry faces was low (Task 1) in participants with high autistic traits. Although the only significant difference in this experiment was in the anger expression, it may be necessary to examine the influence of autistic traits in terms of emotional valence and arousal factors. Anger emotion is characterized by negative emotional valence along with higher arousal. Considering previous studies, the perception of negative emotional valence may have the strongest association with autistic traits [13,14,57]. This is consistent with the pattern of communication reported in people with ASD. For example, one of the features of people with ASD is the difficulty to evaluate others' emotional states. As a result, people with ASD may be unaware when others feel uncomfortable [58]. This communication pattern may rely on the decrease in the perceived intensity of anger emotion in people with ASD.

Contradictory to Hypothesis 2, the effect of autistic traits on arousal and attentional allocations was unstable. AQ scores were entirely not correlated with pupil sizes and viewing times, although the score was correlated with the viewing times for disgusted eyes (Task 1). Therefore, our results do not support the idea that untypical arousal and attentional characteristics mediate the temporal ensembles in individuals with higher autistic traits. For the facial emotion effect on pupil size in people with ASD, there have been contradictory reports. Pupil size in persons with ASD has been reported to remain the same between facial emotions [59], or to have become narrower for emotional expressions (this effect was not observed when the task was repeated [60]). Conversely, Reisinger *et al*. [61] reported that the pupils of people with ASD became enlarged when looking at emotional expressions. The viewing time data were inconsistent with untypical attentional allocations for facial expression [19,20]. The inconsistency may stem from the differences in participants. Although these previous studies recruited individuals with ASD, we studied typically developing individuals and examined the effect of AQ scores. The differences might result in low effects of autistic traits. In line with this idea, we observed a significant interaction between AQ scores and facial emotion on viewing times for a mouth, although *post hoc* analyses showed non-significant effects. Our results did not necessarily indicate that the autistic traits were unrelated to arousal and attentional allocation for facial parts.

This study had three potential limitations. First, we used a simplified procedure to measure the temporal ensembles, which may make it difficult to interpret the results. As noted in the Introduction section, a classic temporal ensemble procedure presents multiple faces that morph with different emotional intensities [9]. By contrast, we used distinct emotional images such as neutral and anger to avoid confounding the effects of temporal parameters and emotional intensity. We measured the perceived intensity of facial emotion. Based on our procedure, it can be argued that the visual difference between neutral and emotional expressions influenced the perceived intensity of facial emotions. To investigate this limitation, future studies should consider manipulating the emotional ratio (or onset timing) while controlling the total intensity of transformed facial expressions. Second, our sample sizes were smaller than those in some previous studies, which also investigated the relationship between autism and facial emotion recognition [62,63]. However, our sample sizes were comparable to those in other previous studies [45–47]. Therefore, we believe that the reliability of our statistical results is not small compared with these previous studies. Third, this study did not include individuals with ASD. Instead, we recruited typically developing people and analysed the effect of the AQ score on the perceived intensity of facial emotions. This limits the application of our results to explain the cognitive characteristics of persons with ASD. Further studies should recruit participants with ASD to examine this topic further.

## 5. Conclusion

The main study results showed that the perceived intensity of emotion was enhanced with a longer emotional expression duration (relative to the total facial presentation duration) and when the emotional expression appeared closer to the moment when participants evaluated the emotion. Moreover, the effects of expression duration and onset timing were lower for the anger perception than the other emotional perception. These results suggested that the facial emotion perception process for changing expressions was influenced by the integration of temporal factors and the types of facial emotions. We also found that the intensity of anger decreased with autistic traits. This finding was consistent with the communication pattern observed among people with ASD.

## Data Availability

Supplemental materials, the raw data and R code for this article are available online at https://osf.io/3tz4s/. The data are provided in electronic supplementary material [64].
